# Using Electronic Health Record Mortality Data to Promote Goals-of-Care Discussions in Seriously Ill Transferred Patients: A Pilot Study

**DOI:** 10.1055/s-0044-1788652

**Published:** 2024-07-24

**Authors:** Neetu Mahendraker, Esmeralda Gutierrez-Asis, Seho Park, Linda S. Williams, Titus Schleyer, Elizabeth E. Umberfield

**Affiliations:** 1 Department of Medicine, Division of General Internal Medicine and Geriatrics, Indiana University School of Medicine, Indianapolis, Indiana, United States; 2 Indiana University Health Physicians Inc., Indianapolis, Indiana, United States; 3 Regenstrief Institute, Indianapolis, Indiana, United States; 4 Department of Industrial and Data Engineering, Hongik University, Seoul, South Korea; 5 Roudebush VA Medical Center Health Services Research and Development, Indianapolis, Indiana, United States; 6 Department of Neurology, Indiana University School of Medicine, Indianapolis, Indiana, United States; 7 Department of Nursing, Division of Nursing Research, Mayo Clinic, Rochester, Minnesota, United States; 8 Department of Artificial Intelligence & Informatics, Mayo Clinic, Rochester, Minnesota, United States

**Keywords:** goals-of-care discussions, inpatient mortality model, mortality risk stratification, clinical decision support, electronic health records

## Abstract

**Background** Mortality prediction data may aid in identifying seriously ill transferred patients at high risk of dying and facilitate early goals-of-care discussions (GOCD); however, this is rarely evaluated. We recently developed a model for predicting 30-day inpatient mortality, which may be useful for promoting early GOCD.

**Objectives** Our objectives were to examine the effects of sharing model-generated mortality risk with hospitalists by assessing (1) if hospitalists agreed with the mortality risk prediction, (2) if they planned to conduct GOCD or consult palliative care within 72 hours of transfer, and (3) if the communication alert affected GOCD timing and other clinical outcomes. We also aimed to measure the association between both the model-generated and hospitalists' stratified risk assessments with patient mortality.

**Methods** This was a nonrandomized quasi-experimental pilot study with a historical control group. On the second day of hospitalization, the model-generated risk was communicated to the hospitalists. Hospitalists were asked to answer questions via a HIPAA (Health Insurance Portability and Accountability Act)-compliant mobile communication system, and clinical outcomes were extracted via chart review.

**Results** Eighty-four patients (42 in the control and 42 in the intervention group) were included in this study. Hospitalists agreed that all patients in the intervention group were at risk for inpatient mortality. Hospitalists were more likely to indicate a plan to conduct GOCD in the intervention group ( *n* = 9) compared with the control group ( *n* = 4, *p* < 0.001). In this subset of patients, GOCD was completed within 72 hours in 78% of intervention patients ( *n* = 7) as compared with 50% in the control group ( *n* = 2). The greater absolute value of the model-generated mortality risk was significantly associated with deaths ( *p* = 0.01), similar to the hospitalists' prediction of the mortality risk ( *p* = 0.02).

**Conclusion** Communicating model-generated mortality risk to hospitalists is a promising approach to promote timely GOCD.

## Background and Significance

**Fig. 1:**
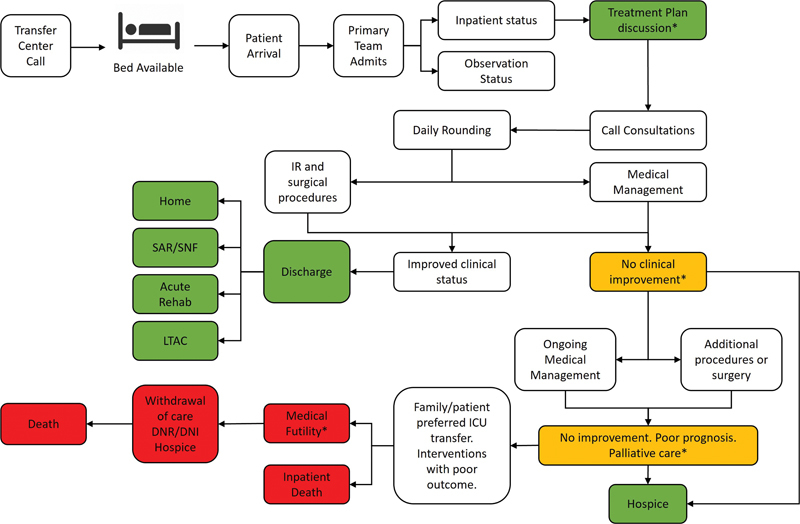
Patient trajectory from an outside facility to Indiana University Health Academic Center leading to discharge or death. *Key decision points are where goals-of-care discussions (GOCD) can occur. The preferred outcome is an early GOCD in the trajectory to provide patient-preferred goal-congruent care. The green box shows preferred outcomes with early GOCD and discharge to the preferred location. The yellow boxes show outcomes that can be improved. The red boxes indicate a poor outcome.

Early referral to palliative and hospice care has been found to improve the quality of end-of-life care and decrease readmission rates, length of hospital stay, and health care costs for seriously ill patients. 1 2 Despite these known benefits, the timing and frequency of palliative care and hospice care consultations vary widely due to their reliance upon clinicians to promptly identify end-of-life care needs. 3 The ability to recognize these needs and conduct goals-of-care discussions (GOCD) is even more challenging for transferred patients at tertiary hospitals due to the lack of continuity of care, advanced patient condition at initial presentation, and the need to discuss sensitive matters in acute crises with little time to establish rapport with patients and families as depicted in Fig. 1 . 4 5 Clinicians have expressed interest in supplementing their clinical judgment with robust clinical prediction models to increase their prognostic confidence, 4 6 7 and strategies have been proposed to aid palliative and hospice care consultations for seriously ill transferred patients with limited life expectancy. 8 9 10 Mortality risk stratification, a systematic technique for categorizing patients' risk of death based on health status and other factors, accompanied by innovative tools to facilitate clinicians' use of assessed risk and guide end-of-life care, is essential for these patients. 11

The rapid growth of data science combined with the wide use of electronic health records (EHRs) allows the timely identification of patients for various purposes using predictive analytics. 12 13 Numerous machine learning models have been developed to predict hospitalized patients' risk of mortality and other adverse health outcomes. 14 15 However, these models are disease-specific, 16 confined to intensive care unit (ICU) patients, exclusively predict postdischarge mortality, 17 or lack prospective evaluation and external validation. 18 19 20 As a result, there is limited evidence that these mortality prediction models could benefit clinician decision-making or improve clinical outcomes in a heterogeneous group of hospitalized patients. A prospective evaluation of the performance of these models using real-world operational data is needed to evaluate their impact on patient care. 16 21 22 23

We recently developed a model that predicts 30-day inpatient mortality among transferred patients based on a retrospective cohort study that examined both administrative and clinical data from 10,389 patients within 24-hour transfer to our medical center. Twenty candidate variables associated with mortality were identified from the EHR. These variables underwent multiple logistic regression and area under the curve-receiver operating characteristic (AUC-ROC) analysis in a derivation sample ( *n* = 5,194) to determine an optimal risk threshold score and develop the model. The final model was validated in a separate sample of patients ( *n* = 5,195), and it demonstrated strong discrimination (C-statistic = 0.90) and good fit. The positive predictive value for 30-day in-hospital death was 68%, with an AUC-ROC of 0.90. A risk threshold score of −2.19 exhibited maximum sensitivity (79.87%) and specificity (85.24%) in the derivation and validation sample (sensitivity: 75.00%, specificity: 85.71%). A complete description of the model's development and evaluation are published elsewhere. 24

In this study, we hypothesized that an intervention involving real-time communication of our model's all-cause 30-day inpatient mortality risk with the primary hospitalists could promote early GOCD in seriously ill transferred patients. 24

## Objectives

Our primary objective was to examine the effects of sharing model-generated mortality risk with hospitalists by assessing (1) if they agreed with the mortality risk, (2) if they planned to conduct GOCD or consult palliative care within 72 hours of transfer, (3) if the communication alert affected GOCD timing and other clinical outcomes. We also aimed to measure the association between both the model-generated and hospitalists' stratified risk assessment with patient mortality.

## Methods

This was a nonrandomized quasi-experimental study incorporating historical controls. The Indiana University Institutional Review Board categorized this study as a quality improvement project under expedited review resulting in waiving the requirement for informed consent. Prior to the study period, we provided an educational session to all hospitalists at the study site about the upcoming pilot and implementation of the mortality risk model. We discussed how the model was developed and shared all the variables that were included in calculating the mortality risk score. 24 All hospitalists provided verbal consent prior to the initiation of this pilot study.

### Clinical Setting

This pilot study was conducted at one of two hospitals in a large, Midwest academic medical center. The academic health center admits about 38,000 patients annually, with about 50% of patients transferred from outside hospitals. The hospitalist service at the study hospital consists of six teams (Red, Blue, Green, Purple, Orange, and Yellow), each with two hospitalists working on a 7-day-on, 7-day-off schedule. As a result, one hospitalist from each team is always present at the facility. Hospitalists on the Red team did not participate as they were primary researchers in this study and Orange team hospitalists could not participate as they were engaged in another study. Concurrent with this study, due to coronavirus disease 2019 (COVID-19) pandemic, the study hospital was designated as the default hospital for patients admitted through the academic medical center's emergency department (ED). This resulted in increasing bed scarcity, more internal admissions, and ultimately fewer external patient transfers. The hospitalist workforce was reinforced with the incremental deployment of locum teams to manage the increased patient census.

### Participants

**Fig. 2:**
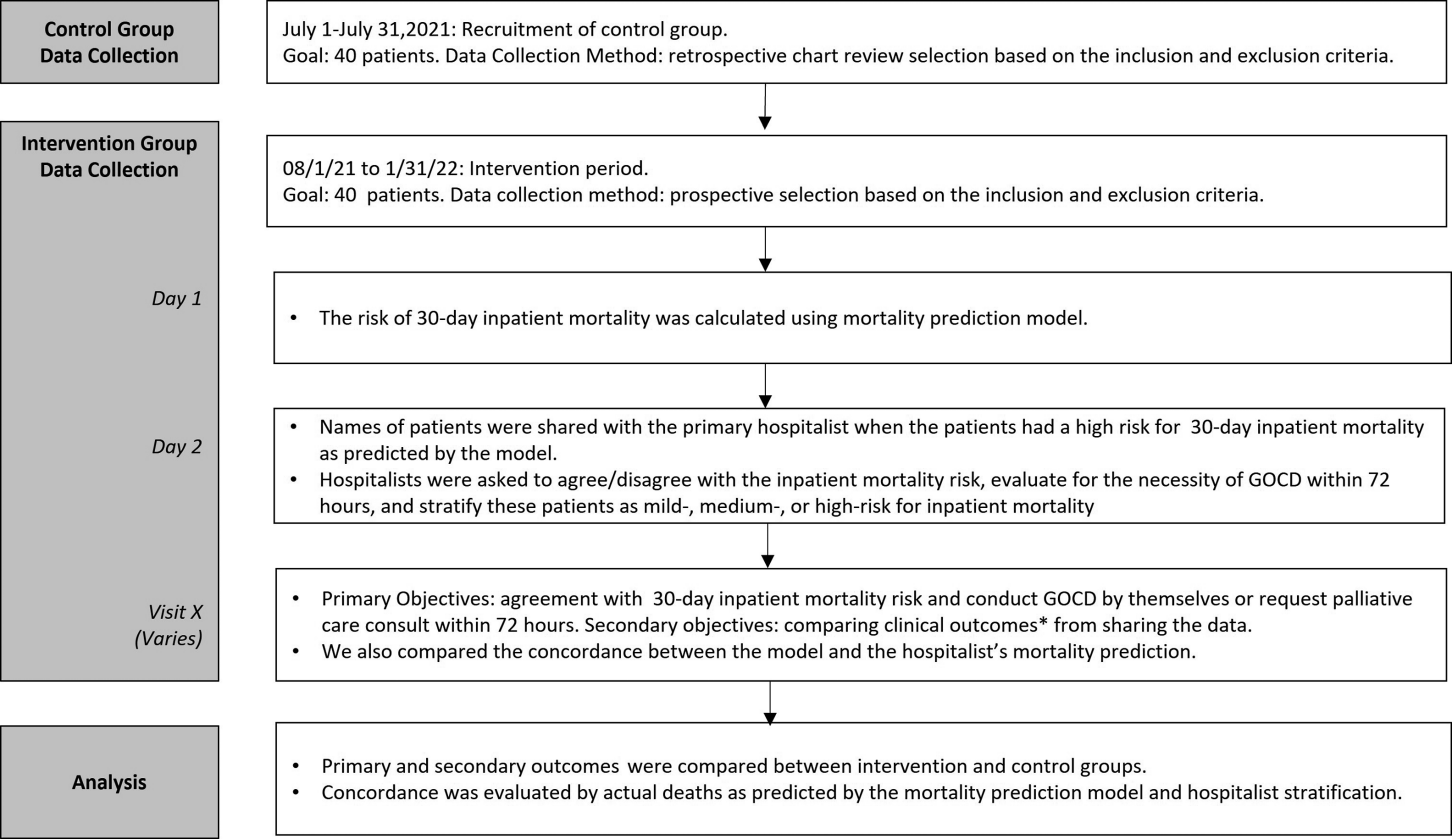
Process of recruitment, data collection, and analysis.

The process of participant recruitment, data collection, and analysis are depicted in Fig. 2 . Patients were eligible for inclusion if they were general medical patients, were 18 years of age or older, transferred from outside facilities, admitted to the hospitalist service, had decision-making capacity or an assigned health care representative/surrogate, and were identified by our model to be at risk for 30-day inpatient mortality. 24 Recruitment was limited to patients admitted between Saturday 1:00 a.m. and Friday 8:00 a.m. during the intervention period to align with our measurement of whether palliative care consultations occurred within 72 hours of admission as there are no palliative care consultations at the hospital over the weekend. Patients were excluded if they were admitted to locum hospitalist teams, if the primary hospitalist teams were primary researcher for this study or if they were involved in concurrent research studies (i.e., Red and Orange teams), if the patients had been transferred directly to our ICU on admission, if the patients were admitted from our ED, if the patients died within 24 hours of transfer, and if they had a documented GOCD or palliative care consultation at an outside hospital before transfer without any changes to the plan of care. We excluded patients who were admitted from our ED as the model was developed specifically for transferred patients and did not include patients admitted directly from our ED. We excluded patients who were admitted directly to our ICU, as our ICU is a closed unit and hospitalists do not have the opportunity to evaluate patients in ICU.

Historical controls were selected to evaluate the intervention's effects within a real-world, nonexperimental context. The historical control group was retrospectively identified among patients transferred to the hospitalist service from July 1, 2021, to July 30, 2021 and met the inclusion and exclusion criteria.

### The Intervention

**Fig. 3:**
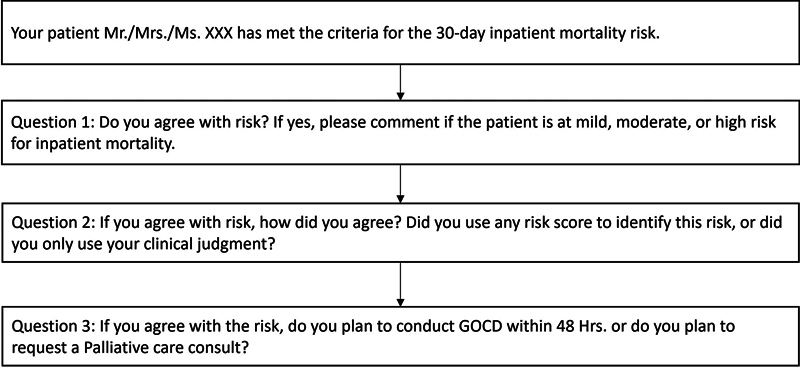
Inpatient mortality risk and GOCD prompt communication to hospitalists in Diagnotes. GOCD, goals-of-care discussion.

The intervention group was recruited from August 1, 2021, to January 30, 2022. Recruitment continued until at least 40 patients or 10 patients from each of the four participating hospitalist teams were reached. Investigators screened eligible patients by reviewing their record and calculating 30-day inpatient mortality risk according to the model 24 within 24 hours of hospitalization. The primary hospitalist was notified on the second day of hospitalization if a patient met the threshold for inpatient mortality risk. Notifications were sent using the HIPAA (Health Insurance Portability and Accountability Act)-compliant mobile communication system, Diagnotes. Fig. 3 presents the initial communication and series of questions asked to hospitalists in Diagnotes. After evaluating the patient, the primary hospitalist answered the series of questions in Diagnotes.

### Data Collection and Management

Data for calculating 30-day mortality risk based on our model were collected by investigators from the EHR. These data were collected, and mortality risk was calculated prospectively within 24 hours of hospitalization for the intervention group and retrospectively for the control group.

The following data were collected from hospitalists via Diagnotes soon after communicating the model's mortality risk: (1) whether they agreed that the patient was at risk for 30-day inpatient mortality risk and stratify that risk as mild, moderate, or high risk; (2) what was their process for determining mortality risk (i.e., did they use a risk score or clinical judgement); and (3) if they planned to offer GOCD or consult palliative care team within 48 hours of receiving the model-predicted high mortality risk communication. Patient outcomes were collected from the EHR via chart review. Incidence of deaths that occurred during the hospitalization or within 30 days of discharge were collected via chart review at 180 days after enrollment in the study to capture any unrecorded deaths at the time of discharge.

Two physician investigators independently reviewed the identified patient records. Interrater reliability was established by comparing data collection between two reviewers and meeting regularly until complete agreement was achieved. All study data were entered into a Microsoft Excel file and saved on an encrypted computer.

### Variables

Demographic variables such as age, sex, and race were examined for a comparative analysis between the control and intervention groups. Additionally, variables measuring social determinants of health, including marital status, employment status, and insurance type, were considered. Body mass index (BMI), a known factor in all-cause mortality, was also a subject of comparison. 25 Baseline characteristics regarding the nature of each patient's hospital admission and stay were also collected. These include where each patient was transferred from, their admission status (inpatient or observation), level of admission (medical–surgical general care or progressive care), and code status on admission.

Primary outcome measures included whether (1) the hospitalists agreed their patients were at risk for 30-day inpatient mortality risk (yes/no), (2) whether the hospitalist planned to conduct GOCD or consult palliative care team within 48 hours of communication (yes/no,) and (3) the number of GOCD conducted within 72 hours of admission.

Secondary clinical outcomes included number of patients with advance directives at discharge, code status at discharge, day of ICU escalation if it occurred, frequency and day of hospice enrollment, frequency and day of readmission within 30 days of discharge, length of stay (i.e., hospitalization during the pilot study), average number of 30-day postdischarge encounters (i.e., readmission or outpatient visit), and frequency of deaths that occurred during the hospitalization or within 30 days of discharge. The impact of communicating model-predicted mortality risk on the timing of initiating a GOCD was recorded in the subgroup of patients in whom hospitalists planned to conduct GOCD or request palliative care consult within 72 hours of admission. Within this subgroup, we recorded the number of patients who actually had GOCD and who conducted the GOCD (i.e., hospitalist or/and palliative care team), by examining documentation of GOCD in the EHR GOCD template or hospitalist progress notes.

### Statistical Analysis

The analysis for this pilot study was primarily descriptive. Comparisons between the intervention and control groups were performed using chi-square tests for categorical variables and a two-tailed *t* -test for continuous variables. To determine the model's and the hospitalists' abilities to predict mortality, model-generated mortality risk score and hospitalists' stratification of mortality risk were compared between patients who were alive and those who died during their hospitalization or within 30 days of discharge. All statistical analyses were performed using SAS software version 9.4 (SAS Institute, Cary, North Carolina, United States), and findings were considered statistically significant at *p* ≤ 0.05.

## Results

A total of 111 patients were screened for inclusion in the study, 84 of whom were eligible and included (42 in each group). Table 1 describes the frequency of baseline patient characteristics among the control and intervention groups and compares these characteristics between groups. Baseline patient characteristics between the control and intervention groups were similar in terms of age, sex, race, marital status, insurance type, admission status, level of admission, and code status on admission. However, patients in the intervention group were more likely to be employed ( *p* = 0.04) and have a lower body mass index ( *p* = 0.001). There were also significant differences among where patients were transferred from ( *p* = 0.01). The model-generated mortality risk score was not significantly different between the control and intervention groups ( *p* = 0.93).

**Table 1:** Comparison of baseline patient characteristics between control and intervention groups a

Characteristic	Control ( *n* = 42)	Intervention ( *n* = 42)	*p* -Value
Age (y), mean (SD)	62.71 (14.61)	68.45 (16.28)	0.09
Sex, female, *n* (%)	20 (47.62)	20 (47.62)	1.00
Race, *n* (%)			1.00
American Indian/Alaskan Native	1(2.38)	1 (2.38)	
Black/African American	3 (7.14)	2 (4.76)	
White	38 (90.48)	39 (92.86)	
Marital status, married, *n* (%)	22 (52.38)	17 (40.48)	0.45
Employment status, employed, *n* (%)	17 (40.48)	6 (14.29)	0.04
Insurance type, *n* (%)			0.09
Medicare/Medicaid	23 (54.76)	34 (80.90)	
Other	19 (45.23)	8 (19.05)	
Body mass index, mean (SD)	30.71 (6.85)	25.87 (6.51)	0.001
Transferred from, *n* (%)			0.01
Outside hospital, emergency department	13 (30.95)	22 (52.38)	0.13
Outside hospital, inpatient	22 (52.38)	8 (19.05)	0.01
Long-term acute care (LTAC)	0 (0.00)	1 (2.38)	NA
Outpatient clinic	5 (11.90)	8 (19.05)	0.41
Home	2 (4.76)	1 (2.38)	0.57
Other (subacute rehabilitation center, prison)	0 (0.00)	2 (4.76)	NA
Admission status, *n* (%)			1.00
Inpatient	41 (97.62)	40 (95.24)	
Observation	1 (2.38)	2 (4.76)	
Level of admission, *n* (%)			0.76
Medical–surgical general care	36 (85.71)	35 (83.33)	
Progressive care	6 (14.29)	7 (16.67)	
Code status on admission, *n* (%)			1.00
Full code	38 (90.48)	39 (92.86)	
DNR/DNI/comprehensive care	4 (9.52)	3 (7.14)	
DNR/DNI/comfort care	0 (0)	0 (0)	
Model-generated mortality risk score, mean (SD)	−0.57 (1.06)	−0.55 (1.06)	0.93

Abbreviation: SD, standard deviation.

a Chi-square testing was used to analyze the categorical data; t-testing was used for continuous data.

Table 2 compares patient outcomes between the control and intervention groups. Hospitalists agreed with the risk of 30-day inpatient mortality as predicted by the model in all patients (100%). Hospitalists indicated the plan to conduct GOCD or consult the palliative care team on day 2 of hospitalization more often in the intervention group than in the control group (21.43 vs. 9.52%, *p* < 0.001). Hospitalists rated 19% of patients in the intervention group as high risk ( *n* = 8), 40% as moderate risk ( *n* = 17), and 40% as low risk ( *n* = 17) for 30-day inpatient mortality based solely on their clinical judgement (i.e., without any clinical decision support tool). Although not statistically significant, our results demonstrate possible patient and family preferred choices in the intervention group including more transitions to DNR/DNI/Comprehensive and comfort care, more enrollments into inpatient hospice, earlier hospice enrollment, more delayed ICU escalations, and fewer inpatient deaths.

**Table 2:** Comparison of patient outcomes between control and intervention groups

Characteristic	Control ( *n* = 42)	Intervention ( *n* = 42)	*p* -Value
Agreed with mortality risk, *n* (%)	–	42 (100)	NA
Hospitalists' mortality risk stratification, *n* (%)	–	42 (100)	NA
High risk	–	8 (19.05)	NA
Moderate risk	–	17 (40.48)	NA
Mild risk	–	17 (40.48)	NA
Hospitalist indicates plan to conduct GOCD or request palliative care consult, *n* (%)	4 (9.52)	9 (21.43)	<0.001
Day of GOCD, mean (SD)	3.25 (2.06)	3.45 (3.42)	0.91
Day of the palliative care consult, mean (SD)	2.33 (2.31)	5.86 (6.57)	0.40
Code status at discharge, *n* (%) a			0.24
Full code	39 (92.8)	33 (78.5)	
DNR/DNI/comprehensive care	1 (2.38)	6 (14.29)	
DNR/DNI/comfort care	2 (4.76)	3 (7.14)	
Escalated to intensive care unit (ICU), *n* (%)	2(4.76)	6(14.29)	0.26
Day of ICU escalation, mean (SD)	5.50 (6.36)	8.50 (9.50)	0.70
Enrolled in hospice, *n* (%)			0.63
Home with hospice	2 (4.76)	3 (7.14)	
Inpatient hospice	1 (2.38)	2 (4.76)	
Day of hospice enrollment, mean (SD)	15.00 (5.66)	9.25 (2.87)	0.15
Advance directives at discharge, *n* (%)	11 (26.19)	11 (26.19)	1.00
Hospital length of stay (d), mean (SD)	8.17 (8.20)	9.76 (7.21)	0.35
30-d readmission, *n* (%)	6 (14.29)	5 (11.90)	0.33
Day of readmission, mean (SD)	11.83 (5.85)	18.40 (9.29)	0.18
30-d postdischarge encounters, mean (SD)	0.66 (0.78)	1.03 (1.04)	0.08
Deceased, *n* (%)	12 (28.57)	9 (21.43)	0.45

Abbreviations: NA, not applicable; SD, standard deviation.

a “Full code” includes performing all available and appropriate resuscitative measures in the event of cardiorespiratory arrest. “DNR/DNI/Comprehensive care” includes standard approach to care but forbids resuscitation (DNR) and intubation (DNI). “DNR/DNI/Comfort care” focuses on providing pain relief and comfort rather than attempting to cure a terminal or serious condition.

Table 3 compares GOCD characteristics in the subgroup of patients in whom hospitalists stated that they planned to conduct GOCD or consult palliative care within 72 hours of admission. A higher rate of GOCD were actually completed within 72 hours among patients in the intervention group than in the control group (75 vs. 50%). The intervention group had a slightly higher proportion of GOCDs conducted by palliative care (78 vs. 50%) and slightly lower proportion of GOCD conducted by hospitalists (33 vs. 50%). When agreed upon and offered, more GOCD were completed within 72 hours in the intervention group.

**Table 3:** Comparison of goals-of-care discussion characteristics between subset of patients in the control and intervention groups whose hospitalists stated that they planned to conduct a goals-of-care discussion

Characteristic	Control ( *n* = 4)	Intervention ( *n* = 9)
GOCD completed in 72 h, *n* (%)	2 (50.00)	7 (78.00)
GOCD by a hospitalist in 72 h, *n* (%)	2 (50.00)	3 (33.33)
GOCD by the palliative care team in 72 h, *n* (%)	2 (50.00)	7 (78.00)

Abbreviation: GOCD, goals-of-care discussion.

Table 4 compares risk score between patients based on their actual mortality. Our results indicate that a greater absolute value of our model-generated mortality risk score was significantly associated with mortality in total sample ( *p* = 0.01), similar to the hospitalists' judgment of mortality risk in the intervention group ( *p* = 0.02).

**Table 4:** Comparison of mortality risk prediction between alive and dead patients

Variable	Alive	Dead	*p* -Value a
Model-generated mortality risk score	( *n* = 63)	( *n* = 21)	0.01
Mean ± SD	−0.4 ± 1.0	−1.1 ± 0.9	
Min–max	−2.0 to 2.2	−2.1 to 0.7	
Hospitalists' risk stratification, *n* (%)	( *n* = 33)	( *n* = 9)	0.02
High risk	3 (4.80)	4 (19.00)	
Moderate risk	13 (20.60)	4 (19.00)	
Mild risk	17 (27.00)	1 (4.80)	

Abbreviation: SD, standard deviation.

a Chi-square testing was used to analyze the categorical data; t-testing was used for continuous data.

## Discussion

This pilot study showed that hospitalists agreed with our 30-day inpatient mortality risk in all patients in the intervention group and that communicating 30-day inpatient mortality risk to hospitalists successfully prompted them to assess these patients for the need for GOCD within 72 hours of admission. Despite the small number of patients in this pilot study, we found that the greater absolute values of the 30-day inpatient mortality risk using our previously developed mortality model 24 were significantly associated with patient death, suggesting this model has validity for future use. Although many of the clinical outcomes in this pilot study did not demonstrate statistically significant effects of the intervention, our results may be clinically meaningful regarding mortality risk communication and earlier transitions to hospice and changes in code status. These changes suggest that without acknowledging these issues, there could be a risk of delivering care that might not align with the patient's and family wishes.

Similar to the model used in a pilot study conducted by Courtright et al, 10 our model has several strengths for real-world applicability, including systematic identification of patients at risk for 30-day inpatient mortality and delivery of actionable information to clinical teams. Unlike the pilot conducted by Courtright et al, 10 our study incorporated physicians' clinical decision autonomy as a first step to risk stratify patients into mild-, moderate-, or high-risk categories and offered hospitalists to opt-in and participate in the initial GOCD with the patient. Use of hospitalists' clinical judgment regarding initiation of GOCD is a strength of our study, as Courtright et al, found that 42.5% of automatically triggered palliative care consultations were declined due to lack of palliative care needs. 10 Although palliative care teams often assist patients and families with GOCD, the palliative care workforce is extremely limited, 26 27 and strategies may be needed to promote palliative care by providers who are not palliative care specialists. .

Another strength of this pilot study is its use of a rigorously developed, internally validated model to identify 30-day inpatient mortality risk, making it more generalizable than previous models. 24 A recent randomized clinical trial by Manz et al had results similar to our pilot study in increasing GOCD 16 but was limited to an outpatient setting and 180-day mortality prediction in oncology patients. Similarly, pilot study by Haley et al 27 examined a narrow patient population, including the factors of cancer, two or more admissions, residence in a nursing home, ICU admission with multiorgan failure, and two or more noncancer hospice guidelines (CARING criteria) to predict 1-year all-cause mortality in hospitalized patients. These criteria were based on group consensus and literature review. 27 In contrast, our focus was mainly on deaths within 30 days of hospitalization, 24 and our model was developed using rigorous analysis of local data. 24

We found that the predictive capabilities of our model's 30-day inpatient mortality risk threshold was comparable to hospitalists' mortality risk prediction. The former employs data-driven algorithms, utilizing diverse clinical variables and historical data to forecast inpatient mortality statistically and may provide an unbiased assessment of numerous factors for a comprehensive outcome. In contrast, the latter relies on medical professionals' expertise, shaped by experience, intuition, and case context. This subjective approach offers nuanced insights yet might be influenced by cognitive biases, limited data access, or overreliance on specific indicators. These differences highlight the contrast between data-driven and expert predictions, which is crucial for precise interpretation and acknowledging their strengths and limitations. 28 Aligned with a recognized pattern identified in prognostication studies among physicians, hospitalists in our study might be predisposed to undervalue the seriousness of their patients' conditions. 29

Our study has several limitations. Generalizability to other patient populations and settings may be limited by small sample size and lack of randomization. Lack of randomization likely contributed to significant differences in baseline characteristics between the intervention and control groups, including employment status, BMI, and where the patient was transferred from. Like Courtright et al, 10 we agreed that alternative study designs, such as randomizing patients at the clinician or unit level, were not feasible for this relatively small pilot study. Measuring goal-congruent care was challenging due to limited advance directives and a lack of standardized GOCD documentation in the EHR. The COVID-19 pandemic, marked by increased patient volume in our ED, limited outside transfers due to bed capacity constraints, and reliance on locum teams, impacted recruitment and prolonged the study duration. Using official death records could have provided more accurate mortality data compared with the EHR data utilized in our study. Our study solely relied on presenting risk data without specifically testing strategies such as participant reactivity and nudge theory. Future research should explore diverse approaches to enhance the effectiveness of interventions targeting clinician behavior. 30 The Hawthorne effect of the GOCD prompt and inherent biases in clinical decision-making tools based on prediction models may have influenced results and decision-making. Moreover, all clinical decision-making tools based on prediction models face the potential of perpetuating human biases present in the foundational data and have the capacity to capture specific practice patterns and the case-mix index at one time point. 23 As with any prediction model, ours may need reevaluation and recalibration to ensure it is clinically meaningful. 31

Despite these limitations, our study was the first step in assessing the impact of prospectively communicating a model-generated mortality risk to hospitalists and evaluating the effects of triggered mortality risk communication on GOCD and patient outcomes. As the next step, we will conduct semi-structured interviews of hospitalists to incorporate their perspectives and preferences to enhance the intervention's acceptability. We also plan to train hospitalists to develop core skills to improve the quality and documentation of GOCD. Hospitalists may consider initiating meaningful GOCD early in the inpatient trajectory ( Fig. 1 ) to optimize end-of-life care and avoid higher health care utilization with burdensome care transitions. 32 33 34

## Conclusion

This pilot study demonstrated promising evidence to support the systematic deployment of our mortality prediction model 23 in seriously ill transferred patients via early communication of the mortality risk with the hospitalists. This intervention may be useful to identify patients at the greatest need for GOCD early in the hospital stay, thus facilitating patient and family preferred end-of-life care. Larger randomized control trials are needed to determine its acceptability and effects on patient outcomes.

## Clinical Relevance Statement

This article describes prospective implementation of a novel clinical decision support system, which alerts hospitalists to risk for 30-day inpatient mortality. In addition, we evaluated the model's performance in clinical practice by assessing the agreement of hospitalists with its recommendations and its impact on GOCD. EHR-based mortality models can provide meaningful input into clinical workflow and decision-making.

## Multiple Choice Questions

1. The electronic health intervention used in this study to identify patients at 30-day inpatient deaths was based on all of the principles below except:
  - Principles of informational nudge.
  - Risk stratification of mortality using EHR data.
  - Augmentation of early clinical decision-making for timely GOCDs by providing patient-specific data.
  - Forbidding a few options that a clinician would otherwise provide if not involved in this study.
  **Correct Answer:** The correct answer is option d. This intervention used the principles of informational nudge that provides information to alter behavior in a predictable way without forbidding any available options. This model risk stratified the 30-day risk for inpatient deaths using EHR-based patient-specific data to augment clinical decision support for timely GOCDs.
2. Most clinicians use their clinical judgment to risk stratify patients for inpatient mortality as:
  - The inpatient mortality models are either disease-specific or limited to intensive care patients and are not generalizable to medical floor patients.
  - Few inpatient mortality models that were proven retrospectively have not been prospectively evaluated in the clinical practice.
  - Risk stratification of mortality prediction is a complex process, and there is not much evidence to support it.
  - If robust mortality prediction models are available, clinicians may be willing to supplement their clinical decisions with the model's input.
  **Correct Answer:** The correct answer is option c. Mortality risk stratification is a complex process and involves several variables, including past medical history, acute presenting condition, hemodynamic stability, vital signs, laboratory results, diagnostic imaging, and response to treatment options. There are insufficient data to conclude that machine learning models are inferior to human mortality risk prediction. Inpatient mortality models that are prospectively evaluated in large, randomized trials and generalizable to all patients admitted and not limited by location or diagnoses are needed as clinicians are willing to adopt such models in their clinical practice.
